# Arguments against the Requirement of a Biological License Application for Human Pancreatic Islets: The Position Statement of the Islets for US Collaborative Presented during the FDA Advisory Committee Meeting

**DOI:** 10.3390/jcm10132878

**Published:** 2021-06-29

**Authors:** Piotr Witkowski, Jon Odorico, Jordan Pyda, Roi Anteby, Robert J. Stratta, Beth A. Schrope, Mark A. Hardy, John Buse, Joseph R. Leventhal, Wanxing Cui, Shakir Hussein, Silke Niederhaus, Jason Gaglia, Chirag S. Desai, Martin Wijkstrom, Fouad Kandeel, Piotr J. Bachul, Yolanda Tai Becker, Ling-Jia Wang, R. Paul Robertson, Oyedolamu K. Olaitan, Tomasz Kozlowski, Peter L. Abrams, Michelle A. Josephson, Kenneth A. Andreoni, Robert C. Harland, Raja Kandaswamy, Andrew M. Posselt, Gregory L. Szot, Camillo Ricordi, on behalf of the Islets for US Collaborative

**Affiliations:** 1 Transplantation Institute, Department of Surgery, University of Chicago, Chicago, IL 60637, USA; Piotr.Bachul@uchospitals.edu (P.J.B.); ybecker@surgery.bsd.uchicago.edu (Y.T.B.); lwang6@bsd.uchicago.edu (L.-J.W.); 2 Division of Transplantation, Department of Surgery, University of Wisconsin, School of Medicine and Public Health, Madison, WI 53792, USA; jon@surgery.wisc.edu; 3 Beth Israel Deaconess Medical Center, Department of Surgery, Harvard Medical School, Boston, MA 02115, USA; jordanpyda@gmail.com; 4 Harvard T.H. Chan School of Public Health, Boston, MA 02115, USA; ranteby@hsph.harvard.edu; 5 Faculty of Medicine, Tel Aviv University, Tel Aviv 69978, Israel; 6 Section of Transplantation, Department of Surgery, Wake Forest School of Medicine, Winston-Salem, NC 27101, USA; rstratta@wakehealth.edu; 7 Department of Surgery, Columbia University College of Physicians and Surgeons, New York, NY 10032, USA; bs170@columbia.edu (B.A.S.); mah1@columbia.edu (M.A.H.); 8 Division of Endocrinology, Department of Medicine, University of NC, Chapel Hill, NC 27516, USA; john_buse@med.unc.edu; 9 Department of Surgery, Northwestern University School of Medicine, Chicago, IL 60611, USA; jleventh@nm.org; 10 Cell Therapy Manufacturing Facility, Georgetown University Hospital, Washington, DC 20007, USA; wanxing.cui@gunet.georgetown.edu; 11 Detroit Medical Center, Department of Surgery, Wayne State School of Medicine, Detroit, MI 48201, USA; shakirhussein00@yahoo.com; 12 Department of Surgery, University of Maryland School of Medicine, Baltimore, MD 21201, USA; sniederhaus@som.umaryland.edu; 13 Joslin Diabetes Center, Harvard Medical School, Boston, MA 02215, USA; jason.gaglia@joslin.harvard.edu; 14 Department of Surgery, Section of Transplantation, University of NC, Chapel Hill, NC 27516, USA; chirag_desai@med.unc.edu; 15 Department of Surgery, University of Pittsburgh, Pittsburgh, PA 15260, USA; wijkstrommn@upmc.edu; 16 Department of Translational Research and Cellular Therapeutics, Diabetes and Metabolism Research Institute, Beckman Research Institute of City of Hope, Duarte, CA 91010, USA; fkandeel@coh.org; 17 Division of Endocrinology and Metabolism, Department of Internal Medicine, University of Washington, Seattle, WA 98133, USA; rpr@pnri.org; 18 Department of Surgery, Rush University Medical Center, Chicago, IL 60612, USA; Oyedolamu_Olaitan@rush.edu; 19 Division of Transplantation, Department of Surgery, The University of Oklahoma College of Medicine, Oklahoma City, OK 73104, USA; tomasz-kozlowski@ouhsc.edu; 20 MedStar Georgetown Transplant Institute, Washington, DC 20007, USA; peter.l.abrams@gunet.georgetown.edu; 21 Department of Medicine, University of Chicago, Chicago, IL 60637, USA; mjosephs@medicine.bsd.uchicago.edu; 22 Department of Surgery, University of Florida, College of Medicine, Gainesville, FL 32610-0118, USA; kenneth.andreoni@gmail.com; 23 Case Western Reserve University, Cleveland, OH 44106-5047, USA; 24 Department of Surgery, University of Arizona, Tucson, AZ 85711, USA; harlandr@surgery.arizona.edu; 25 Department of Surgery, University of Minnesota, Minneapolis, MN 55455, USA; rk1@umn.edu; 26 Division of Transplantation, Department of Surgery, University of California San Francisco, San Francisco, CA 94143, USA; andrew.posselt@ucsf.edu (A.M.P.); Gregory.Szot@ucsf.edu (G.L.S.); 27 Diabetes Research Institute and Cell Transplant Center, University of Miami, Miami, FL 33136, USA; cricordi@med.miami.edu

**Keywords:** allogenic islet cell transplantation, pancreas, Food and Drug Administration

## Abstract

The Food and Drug Administration (FDA) has been regulating human islets for allotransplantation as a biologic drug in the US. Consequently, the requirement of a biological license application (BLA) approval before clinical use of islet transplantation as a standard of care procedure has stalled the development of the field for the last 20 years. Herein, we provide our commentary to the multiple FDA’s position papers and guidance for industry arguing that BLA requirement has been inappropriately applied to allogeneic islets, which was delivered to the FDA Cellular, Tissue and Gene Therapies Advisory Committee on 15 April 2021. We provided evidence that BLA requirement and drug related regulations are inadequate in reassuring islet product quality and potency as well as patient safety and clinical outcomes. As leaders in the field of transplantation and endocrinology under the “Islets for US Collaborative” designation, we examined the current regulatory status of islet transplantation in the US and identified several anticipated negative consequences of the BLA approval. In our commentary we also offer an alternative pathway for islet transplantation under the regulatory framework for organ transplantation, which would address deficiencies of in current system.

## 1. Introduction

The Food and Drug Administration (FDA) decided to regulate human islets for transplantation as biological drugs in the United States (US) and published several position papers and a guidance for industry [1,2,3,4]. Consequently, human islet safety and effectiveness has been confirmed in clinical trials, but a subsequent requirement of the biological license application (BLA) approval before clinical use of islet transplantation has stalled the development of the field for the last 20 years. However, a BLA for human islets has been recently submitted by a private for-profit entity (BLA 125734). If approved, the FDA will effectively allow the commercialization of human islets that will be used for transplantation. This is unprecedented in the field of organ transplantation, where the sale of kidneys, for example, is at least unethical and at most, a criminal offense. 

Herein, we provide our commentary to the FDA position arguing that BLA requirement has been inappropriately applied to allogeneic islets. As a group of experts and leaders in the field of transplantation and endocrinology (Islets for US Collaborative) we strongly advise against the BLA approval and have warned of the negative consequences this would have for patient safety, as well as the field of islet transplantation. We also propose a regulatory path for human islets similar to the one that already exists for other human organs in the US where oversight would be provided by the Health Resources and Services Administration (HRSA) along with the Organ Procurement and Transplantation Network (OPTN) and the United Network for Organ Sharing (UNOS) [5,6]. However, the FDA recently rejected this proposal, and the BLA approval is imminent [7,8]. 

The Islets for US Collaborative comprises more than 50 medical experts and leaders in the fields of transplantation and diabetes from leading US academic institutions who have longstanding concerns about the regulatory status of islet transplantation in the US (www.isletsforus.org). Our intention is to share our concerns and recommendations with the broad medical community, government, and regulatory officials as well as our patients, and hopefully stimulate considerations of appropriate regulatory adjustments. 

## 2. Position against Regulation of Human Islets as Drugs and BLA Approval

We question whether the BLA for human islets should be approved, as it raises significant ethical, legal, policy, and public health considerations that should properly be addressed by the Secretary of Health and Human Services (HHS). 

Human pancreatic islets are isolated from the deceased donor pancreas and transplanted into the recipient’s liver. Islet transplant recipients require the same complex medical therapy and immunosuppression medications as any other patients receiving organ transplantation. Here, we present several arguments against regulation of human islets as drugs indicating rather the need for regulation designed for human organs for transplantation in the US.

### 2.1. Islets as Organs

**Figure 1:**
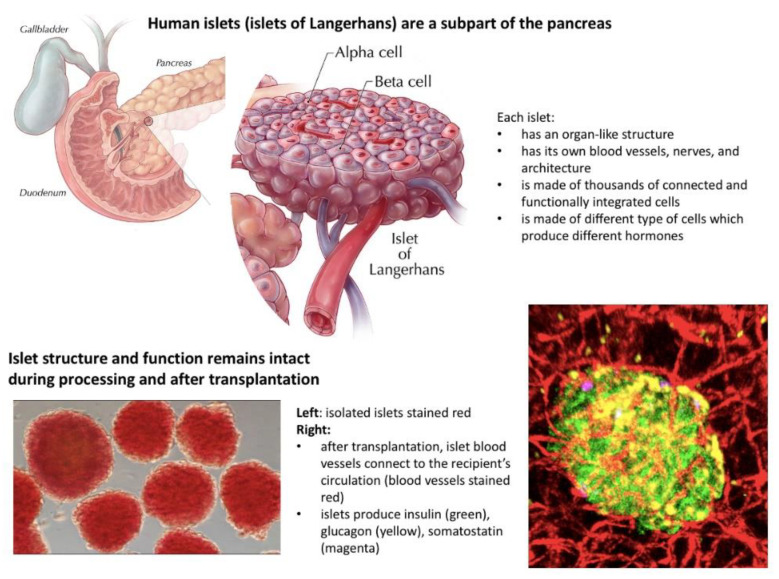
Isolation and processing of human pancreatic islets for transplantation. Images of islets are provided courtesy of Dr. Manami Hara from the University of Chicago. The commonly used term “islet cell transplantation” erroneously suggests that: (1) islets disintegrate into a mixture of single cells during processing prior to transplantation; (2) are “more than minimally manipulated”; and (3) need to be regulated as biologic drugs. However, islets are micro-organs and remain intact during pre-transplantation processing, and they are not more than minimally manipulated. Moreover, after transplantation, islets form new vascular connections and integrate with the recipient’s circulation.

Islets are human micro-organs and should be regulated consistent with the pancreas and other human organs, which are not regulated by the FDA in the US and for which BLAs are not required [4,5] (Figure 1).

Islets are not drugs, and they are not a form of cellular therapy. Islets, like any other organ for transplantation, encompass the following characteristics [4,5]:

- Exist naturally in the human body and are not artificially manufactured;
- Consist of many different types of cells with unique and well-integrated functions;
- Have their own internal blood vessel and neural network;
- Maintain their own morphology and structure during processing and after transplantation;
- Connect their own vasculature to the recipient blood vessel network after transplantation;
- Do not tolerate below zero temperatures and can be preserved in room temperature for only a short period of time;
- Most importantly, in contrast to drugs, the potency of islets as with any other organ for transplantation cannot be reassured by a single test prior to transplantation but can be reassured only by the continuous assessment by the transplant team of complex parameters and constant supervision from the moment of donor selection through pancreas recovery, processing, preservation, transplantation, and, finally, post-transplant patient care in order to provide safe, effective, and appropriate clinical care.

Altogether, islets, as with any other organ and in contrast to drugs, cannot be stored in commercial organ banks and have a potency certificate for use in transplantation. Organ and islet potency can only be verified after the transplantation based on successful clinical outcomes. For this reason, organ transplant programs are held accountable for the outcomes of islet transplantation. 

Human islets, as with other organs, are naturally highly variable, and thus cannot be standardized as drugs. Given the above-mentioned reasons, islets, as with other organs for transplantation, have not and will not fit into the frame of drug regulations. As a result, safety and effectiveness cannot be reassured by drug-specific regulations. However, islets as organs for transplantation do benefit patients when they are transplanted in a proper setting with proper clinical oversight. 

### 2.2. The FDA’s Position That Allogeneic Islets Are Drugs and Require a BLA Has Prevented Islet Transplantation from Becoming a Standard of Care Procedure in the US, in Contrast to Other Countries

Several academic transplant centers in the US have successfully processed human islets for transplantation in clinical trials without a BLA over the last 20 years. However, transplant centers are not drug manufacturers, and are not in a position to sponsor a BLA or comply with the FDA’s other drug manufacturing requirements. BLA submissions are not aligned with the mission of academic transplant centers that lack appropriate organizational structure and resources, making it extremely difficult, if not impossible, to meet financial and legal BLA demands. Nor are such requirements necessary for safe and effective islet transplantation.

**Figure 2:**
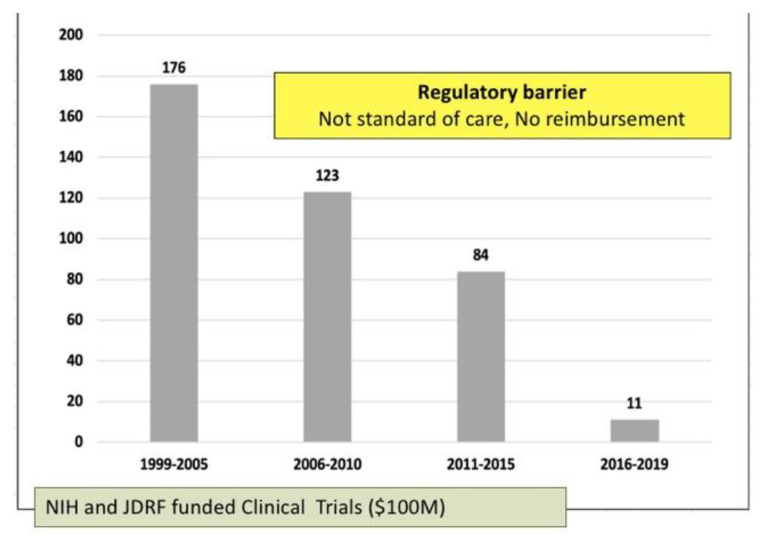
Catastrophic decline of allogenic islet transplantation in the US. Graph courtesy of Dr. Franca Barton from Collaborative Islet Transplantation Registry, USA.

Consequently, after 20 years of research and clinical trials, islet transplantation is still not broadly available to Americans with Type 1 Diabetes. Over the last 5 years, the number of patients treated with islet transplantation dropped to only a few per year nationally as depicted in the figure below (Figure 2).

Inversely, in Canada, Australia, Japan, and throughout Europe [9], islets are regulated as organs or tissue for transplantation, and these regions have already adapted clinical islet transplantation as a standard of care procedure for patients. In fact, these programs have directly benefited from American led research on islet isolation and transplantation technology, which was made possible through millions of dollars of federal research funds. 

### 2.3. Granting a BLA to a Private, For-Profit Company Will Not Solve the Problem of Islet Transplantation in the US But Will Lead to Its Further Demise

If the BLA is approved, a for-profit entity will have the following privileges:

- A right to commercialize human islets as a biological drug for use in transplantation. This is inconsistent with the federal prohibition on commercialization of human organs [3];
- 7 years of marketing exclusivity under the Orphan Drug Act;
- Significant leverage in terms of the contract with any transplant center to provide islets for transplantation, including the price for islets;
- Unprecedented influence over which transplant centers are able to offer their patients islet transplantation.
- As such, we foresee the following downstream consequences:
- Transplant centers will have no alternative source of islets for clinical use;
- Transplant centers will have no control over the quality of islets for their patients;
- Access to islet transplantation will be limited due to increased costs and decreased availability of islets compared to a situation where islets are regulated as organs and are exempt from BLA.

### 2.4. Granting This BLA May Compromise Patient Safety

The Health Resources and Services Administration (HRSA) developed regulations to ensure the safe and ethical allocation and transplantation of human organs in the US. Under the HRSA, the Organ Procurement and Transplantation Network (OPTN) and United Network for Organ Sharing (UNOS) oversees academic medical center transplant programs that provide complex medical therapy through a multidisciplinary team of transplant physicians. The OPTN/UNOS oversight framework is critical to assure the safety and effectiveness of the therapy, which extends beyond the transplantation procedure. 

As a result of the BLA requirement, islets will be excluded from the OPTN/UNOS oversight. Thus, the commercial BLA holder and patients after islet transplantation will not be subject to OPTN/UNOS post-transplant monitoring of patient outcomes. As a result, islets will be less regulated than all other human organs.

### 2.5. Proposed Solution 

As a solution we propose human pancreatic islets be regulated as organs by HRSA through OPTN and UNOS, and not as drugs by the FDA. The National Organ Transplantation Act defines a human organ to include both whole organs and subparts of organs (42 USC § 274e(c)(1) amended in 1988). There is no dispute that islets are subparts of the pancreas, and therefore should be regulated as human organs and should not be subjected to a BLA. 

However, to make this happen, HRSA regulations (the OPTN Final Rule) need to be updated so that the regulatory definition of human organ matches the statutory definition. Because of the mismatch between the statute and regulations, for the past 20 years, the FDA has taken the position that islets are a biological drug requiring a BLA, with negative downstream consequences for the field of islet transplantation.

We call upon the Secretary of HHS to designate allogeneic islets for transplantation as human organs under the OPTN Final Rule. This will achieve the following:

- Legally, it would conform with the statutory definition of human organs under the National Organ Transplantation Act (NOTA);
- Provide OPTN/UNOS with legal authority for holistic, systematic clinical oversight over islet transplantation. Thus, protecting patients by ensuring the safety and effectiveness of islet transplantation therapy;
- Prevent imminent commercialization of human islets, which is prohibited under NOTA, by preventing the FDA from granting a BLA for human islets to a commercial entity.

The HHS Secretary’s decision in 2013 to include vascularized composite allografts (VCAs) under OPTN/UNOS, rather than FDA jurisdiction, was stimulated by the same organ-like nature and safety rationale and provides a strong precedent for including human islets under the OPTN final rule. We believe that a solution that regulates islets as an organ rather than a drug would not compromise islet processing regulatory oversight, which could remain subject to FDA Good Tissue Practice (GTP) requirements as currently is the case for islets for autologous use processed in the same manner as islets for allogeneic use.

### 2.6. FDA’s Position That a BLA Is Required for Unrelated Allogeneic Islets Is Inconsistent with the Agency’s Approach to Autologous Islets

In cases where the islets are for use in the same person (autologous transplant), the FDA does not require a BLA, and subsequently drug manufacturing conditions are not required for islet processing (Section 361 of the Public Health Service (PHS) Act). Furthermore, if islets are for use in first-/second-degree relatives (allogeneic transplant), no BLA is needed, and no drug manufacturing conditions are required for islet processing (Section 361, PHS Act). 

However, if islets are used between unrelated people (allogeneic transplant), BLA and drug related regulations (Section 351, PHS Act) are indeed required by the FDA. This is preposterous, given that the same islet isolation technique is used for allogeneic and autologous islets, but only unrelated allogeneic islets require a BLA.

**Figure 3:**
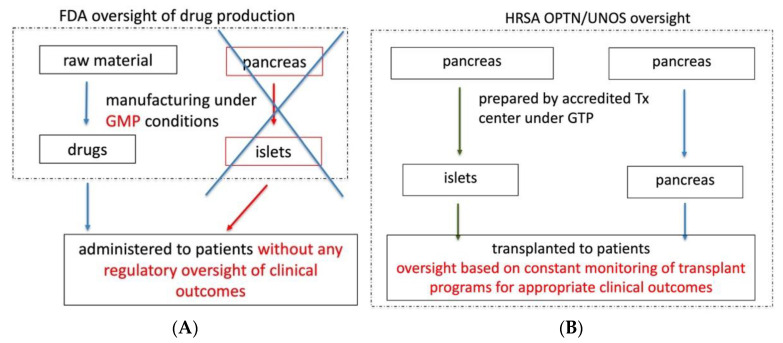
A proposed framework for Islet cell regulations in the US. Application of drug manufacturing regulations does not provide appropriate regulatory oversight of patient care and clinical outcomes (**A**). OPTN/UNOS constantly monitor transplant programs for appropriate clinical outcomes as a condition for maintaining the accreditation. Outcomes are also under public scrutiny and available on the UNOS public website (**B**).

We believe that the FDA is disadvantaging patients who would benefit from unrelated allogeneic islets without an apparent safety rationale for imposing additional drug manufacturing standards, which do not provide regulatory oversight of clinical use of islets. It is not the FDA, but the OPTN/UNOS regulations, that provide an appropriate regulatory framework for the clinical use of allogeneic islets and assure safety and effectiveness. Therefore, allogeneic islets should be regulated as organs, not as drugs (Figure 3). 

Appropriately regulating islets under OPTN/UNOS protection will allow academic centers to continue to process and transplant human islets. This maintains the field under healthy academic competition, whilst stimulating progress and access to islet transplantation.

In March of 2021 we sent a letter to the Secretary of Health and Human Services containing arguments presented in this commentary pointing out the need for an urgent regulatory update for allogenic islets in the United States. 

## 3. Conclusions

Approval of the Biological License Application will result in adverse, potentially irreversible, downstream consequences for patient safety and limit access to the effective islet transplantation procedure. We call the FDA, HRSA, and the Secretary of the HHS to reconsider appropriate adjustments in the regulation for the benefit of enhancing the health and well-being of Americans with Type 1 diabetes mellitus and progress in the field of islet transplantation. 

## Data Availability

Not applicable.

## Supplementary Materials

The following are available online at https://www.mdpi.com/article/10.3390/jcm10132878/s1, Title: The Islets for US Collaborative.
