# Supplementary material for: Arguments against the Requirement of a Biological License Application for Human Pancreatic Islets: The Position Statement of the Islets for US Collaborative Presented during the FDA Advisory Committee Meeting
Source: J Clin Med. 2021 Jun 29;10(13):2878. doi: 10.3390/jcm10132878 (PMC8269003; doi:10.3390/jcm10132878)
Supplement: Supplementary file 1 [file jcm-10-02878-s001.zip › jcm-1244666-supplementary.pdf]

The “Islets for US” Collaborative

Experts

Piotr Witkowski, MD, PhD	University of Chicago, Chicago, IL
Camillo Ricordi, MD	University of Miami, Miami, FL

American Society of Transplant Surgeons (ASTS)

Marwan S. Abouljoud, MD	President	Henry Ford Hospital, Detroit, MI
Kenneth Andreoni, MD	Council-At-Large	University of Florida, Gainesville, FL
Robert Harland, MD	Cellular Transplantation Committee	University of Arizona, Tucson, AZ
Lloyd Ratner, MD	Immediate Past President	Columbia University, New York, NY
Dixon B. Kaufman, MD PhD	Past President	University of Wisconsin, Madison, WI
Peter Stock, MD PhD	Past President	UCSF, San Francisco, CA
Mark A. Hardy MD	Past President	Columbia University, , New York, NY
Jason Wellen, MD		Washington University, St. Louis, MI
Peter L. Abrams, MD		MedStar Georgetown, Washington, DC
Marlon Levy, MD		Virginia Commonwealth, Richmond, VA
Chirag S. Desai, MD		Washington University, St. Louis, MI
Michael Millis, MD		University of Chicago, Chicago, IL
Robert J. Stratta, MD		Wake Forest, Winston-Salem, NC
Jonathan A. Fridell, MD		Indiana University, Indianapolis, IN
Tomasz Kozlowski, MD		Oklahoma University, Oklahoma City, OK
Rolf N. Barth, MD		University of Chicago, Chicago, IL
Piotr J. Bachul, MD		University of Chicago, Chicago, IL
Jordan S. Pyda, MD, MPH		Harvard Medical School, Boston, MA
Kumar Jayant, MD		University of Chicago, Chicago, IL
Martin Wijkstrom MD		University of Pittsburgh, Pittsburgh, PA
Joseph Leventhal MD PhD		Northwestern University, Chicago, IL
Shakir Hussain, MD		Detroit Medical Center, Detroit, MI
Abbas Rana, MD		Baylor Medical College, Houston, TX
Andrew Posselt MD		UCSF, San Francisco, CA

American Diabetes Association (ADA),
Experts in Diabetology and Islet Transplantation

Louis Philipson, MD, PhD	Immediate Past President,	University of Chicago, Chicago, IL,
John Buse, MD, PhD	Past President	University of North Carolina, Chapel Hill
R. Paul Robertson MD	Past President	University of Washington, Seattle, WA
Rodolfo Alejandro, MD		University of Miami, Miami, FL
David Baidal, MD		University of Miami, Miami, FL
Melena Bellin, MD,		University of Minnesota, Minneapolis, MN
Fouad Kandeel, MD, PhD		City of Hope, Duarte, CA
Jason Gaglia, MD		Harvard Medical School, Boston, MA
Raghavendra G. Mirmira, MD, PhD		University of Chicago, Chicago, IL

International Pancreas and Islet Transplantation Association

James F. Markmann, MD, PhD	President	Harvard Medical School, Boston, MA
Raja Kandaswamy, MD	President-Elect	University of Minnesota, Minneapolis, MN
Jon Odorico, MD,	Past President	University of Wisconsin, Madison, WI

United Network for Organ Transplantation (UNOS)

David Mulligan, MD	President	Yale University, New Haven, CT
Yolanda Becker, MD	Immediate Past President	University of Chicago, Chicago, IL
Silke Niederhaus, MD,	Chair of Pancreas ad Islet Tx Committee	University of Maryland, Baltimore, MD
Rachael C. Forbes, MD	Vice Chair of Pancreas ad Islet Tx Committee	Vanderbilt University, Nashville, TN
Oyedolamu K. Olaitan, MD	Pancreas ad Islet Tx Committee	Rush University, Chicago, IL

American Society of Transplantation

Michelle A. Josephson, MD		University of Chicago, Chicago, IL
Ling-Xin Chen, MD		UC Davis, Sacramento, CA
Michael Charlton, MD		University of Chicago, Chicago, IL
Xunrong Luo, MD PhD		Duke University Durham, NC

The Transplantation Society

John Fung, MD, PhD	Council	University of Chicago, Chicago, IL

American College of Surgeons

Beth Schrope, MD		Columbia University, New York, NY

Experts in Cellular Therapy

Yossi Schwartz, MD	Secretary , World Apheresis Association	Columbia University, New York, NY
Amittha Wickrema, PhD		University of Chicago, Chicago, IL
Wanxing Cui, MD PhD		Georgetown University, Washington DC

Islet Processing Experts

Karolina Golab, PhD		University of Chicago
Appakalai N. Balamurugan, PhD		University of Cincinnati, Cincinnati, OH
Greg Szot, MS		UCSF, San Francisco, CA

Drugs, Biologics, and Medical Device Development Advisor

Anthony J. Japour, MD — Anthony Japour & Associates, Medical and Scientific Consulting, Miami

Legal Expert (FDA Regulations)

Gail Javitt, JD, MPH — John Hopkins University, Baltimore, MD

M Eryk Nowicki JD — SWC Nowicki, Chicago, IL

SUPPORTERS

"Your brilliant research ought not to be kept from US patients", September 1, 2019, STAT NEWS,

commentary to Dr. Ricordi's article "Transplanting islet cells can fix brittle diabetes. Why isn't it available in the U.S.?

Donna Shalala

Congresswoman, past President of University of Miami and the Clinton Foundation

The longest-service US Secretary of Health and Human Services in American history (1993-2001)

The Cure Alliance non-profit organization helping accelerate potential cures from the laboratory to the bedside
